# Wide-Field Swept-Source OCT Analysis of Interocular Symmetry of Choroidal Thickness in Subjects with Uncomplicated Pachychoroid

**DOI:** 10.3390/jcm10184253

**Published:** 2021-09-19

**Authors:** Min-Su Kim, Hyung-Bin Lim, Woo-Hyuk Lee, Yeo-Kyoung Won, Ki-Yup Nam, Jung-Yeul Kim

**Affiliations:** 1 Department of Ophthalmology, Chungnam National University College of Medicine, Daejeon 35015, Korea; kms1406@naver.com (M.-S.K.); cromfans@hanmail.net (H.-B.L.); wyk900105@hanmail.net (Y.-K.W.); 2 Department of Ophthalmology, Gyeongsang National University College of Medicine, Changwon 51472, Korea; lwhyuk@naver.com; 3 Department of Ophthalmology, Chungnam National University College of Medicine, Sejong 30099, Korea; oksnam1231@hanmail.net

**Keywords:** choroidal thickness, interocular symmetry, uncomplicated pachychoroid, wide-field swept-source optical coherence tomography

## Abstract

Background: We aimed to study the bilateral choroidal thickness (CT) symmetry and difference in uncomplicated pachychoroid subjects using wide-field swept-source optical coherence tomography (SS-OCT). Methods: All subjects underwent a wide-field 16-mm one-line scan using SS-OCT. Bilateral CT was measured at, and compared among, the following 12 points: three points at 900-µm intervals from the nasal optic disc margin (nasal peripapillary area), one point at the subfovea, six points at 900-µm intervals from the fovea to the nasal and temporal areas (macular area), and two peripheral points 5400 and 8100 µm from the fovea (peripheral area). Results: There were no statistically significant differences in CT between the right and left eyes in any area (all *p* > 0.05); they all showed significant positive correlations (all *p* < 0.01). However, the correlation coefficients (ρ) were smaller for the nasal peripapillary and peripheral areas compared to the macular area. Conclusions: The CTs in each region were bilaterally symmetrical in subjects with uncomplicated pachychoroid. However, interocular difference in CT increased from the center to the periphery, indicating that the anatomical variation of the nasal peripapillary and peripheral choroid was greater than that of the macula.

## 1. Introduction

The rapid development of optical coherence tomography (OCT) has shed light on the morphological and pathophysiological features of various chorioretinal diseases [1]. In particular, enhanced depth image (EDI)- and swept source (SS)-OCT enable more accurate qualitative and quantitative analyses of the choroid than conventional SD-OCT [2,3,4]. Detailed analysis of the choroid using these state-of-the-art imaging techniques has led to new concepts, such as “pachychoroid” and “pachychoroid disease spectrum”.

The pachychoroid disease spectrum, first introduced by Warrow et al. [5], is characterized by increased focal or diffuse choroidal thickening, a pathologically dilated vein in Haller’s layer (pachyvessel) and thinning in Sattler’s layer and the choriocapillary layer [6,7,8]. The pachychoroid disease spectrum includes pachychoroid pigment epitheliopathy (PPE), central serous chorioretinopathy (CSC), pachychoroid neovasculopathy (PNV), polypoidal choroidal vasculopathy (PCV), focal choroidal excavation, and peripapillary pachychoroid syndrome [7]. An abnormally thick choroid without these specific findings on retinal imaging is referred to as uncomplicated pachychoroid [7,8,9,10]. Several studies have proposed cutoff choroidal thickness (CT) values to define a thick choroid. However, CT can be affected by various factors, including age [11,12], sex [13], axial length (AL) [13,14], spherical equivalent (SE) [11], intraocular pressure (IOP) [15], mean arterial pressure [16], and diurnal variation [17]. There is no consensus regarding the cutoff value for thick choroid, but many studies defined pachychoroid as CT > 300 μm [7,10,18].

The recently developed swept-source OCT (SS-OCT) uses a wavelength of 1040–1060 μm, which enables deeper penetration. Therefore, SS-OCT can be used to obtain more detailed and clearer images of deeper structures (e.g., the choroid and choroidoscleral junction) than previous imaging modalities. The scan rate of commercially available SS-OCT is nearly twofold higher than that of conventional SD-OCT, thus reducing motion artifacts and enabling acquisition of wide-field B-scan images [19]. A number of studies on peripheral retinal and choroidal morphology have been conducted using wide-field OCT [20,21,22].

In most healthy individuals, both eyes are not anatomically or functionally identical, but have a generally similar appearance. Therefore, if there is a change in interocular symmetry, the physician should seek to determine whether it is due to disease or constitutes asymmetry within the normal range, as this has important implications for treatment planning. To our knowledge, there have been no studies on the interocular symmetry of CT in uncomplicated pachychoroid. In this study, we compared bilateral CT values among the macular, nasal peripapillary, and peripheral areas using wide-field (16-mm) SS-OCT in subjects with uncomplicated pachychoroid.

## 2. Materials and Methods

This retrospective, observational study was approved by the Institutional Review Board of Chungnam National University Hospital (Daejeon, Republic of Korea). Informed consent was obtained from all participants, and the study protocol adhered to the tenets of the Declaration of Helsinki.

### 2.1. Participants

The study population consisted of young, healthy adults with uncomplicated pachychoroid, all of whom visited the retina clinic of Chungnam National University Hospital for retina and vitreous evaluation between March 2018 and June 2020. Information on age, sex, medical history, and history of previous ocular surgery were collected; all subjects underwent comprehensive assessments of best-corrected visual acuity (BCVA), IOP (CT-80; Topcon Corporation, Tokyo, Japan), SE (KR-1; Topcon Corporation), and AL (IOL Master^®^; Carl Zeiss Meditec, Jena, Germany), as well as dilated fundus examinations. SS-OCT (PLEX Elite 9000; Carl Zeiss Meditec, Dublin, CA, USA) was performed to evaluate baseline ocular findings and measure CT. As in previous studies [7,8,9,10,18], uncomplicated pachychoroid was defined as eyes with thick choroid (subfoveal CT > 300 μm or and extrafoveal focus that exceeded subfoveal CT by at least 50 μm), pachyvessels (dilated choroidal vessels), inner choroidal attenuation, and no abnormal findings (e.g., PPE, CSC, PNV, and PCV) on OCT imaging. We also defined pachyvessels as dilated outer choroidal vessels observed on SS-OCT en face slabs of outer choroid, which correlated with the areas of maximal CT with increased Haller’s layer in cross-sectional OCT.

This study included subjects with bilateral uncomplicated pachychoroid and BCVA of 20/20 or better, none of whom had any medical history (e.g., diabetes or hypertension). Subjects with unilateral uncomplicated pachychoroid, SE < −6.0 D, AL > 26.5 mm, anisometropia > 3.0 D, IOP > 21 mmHg, chorioretinal disease, glaucoma, optic nerve disease, or previous ocular surgery history (including refractive surgery) were excluded.

### 2.2. Image Acquisition

The Zeiss PLEX^®^ Elite 9000 instrument is based on SS-OCT and uses a swept-source tunable laser with a center wavelength between 1040 nm and 1060 nm as a light source. In addition, it has a speed of 100,000 A-scans/s and, in tissue, provides an A-scan depth of 3.0 mm, an optical axial resolution of 6.3 μm, a digital axial resolution of 1.95 μm, and a transverse resolution of 20 μm.

The Zeiss PLEX^®^ Elite 9000 instrument offers a variety of scan types. In this study, the HD spotlight 1 (16 mm) (magnification, 10×–100×) scan was used. This provides a single, high-definition scan with a depth of 3.0 mm, 100 B-scans, 1024 A-scans, and a length of 16 mm anywhere on the fundus image. The examiner can set the number of scan frames (scan repetitions) to 10–100 (10-scan interval). In this study, the HD spotlight 1 scan (length of 16 mm, 100 scan frames) set the scan angle to 0 degrees to take a horizontal scan including fovea, and it was performed twice for all participants by an experienced examiner. The best scan with a signal strength ≥9 was selected for the analysis. The results of individuals with an OCT scan signal strength <9 or scan artifacts were excluded.

### 2.3. CT Measurements

**Figure 1:**
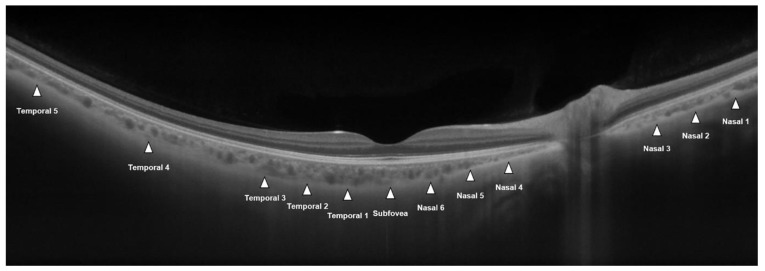
A 16-mm wide-field (16-mm) optical coherence tomography image of a healthy individual. Choroidal thickness was measured with a caliper at 12 points, i.e., at 900-µm intervals from the nasal optic disc margin (nasal points 1–3), at the subfovea, at 900-µm intervals from the fovea (nasal points 4–6 and temporal points 1–3), and at 2700-µm intervals from temporal point 3 (temporal points 4 and 5).

CT measurements were conducted in the same manner as described in our previous report [23]. For the 16-mm HD spotlight scan, CT was measured from the outer part of the hyperreflective line (corresponding to the retinal pigment epithelium RPE) to the inner surface of the sclera, using a caliper and built-in review software. Measurements were made at 12 points: 3 points at 900-µm intervals from the nasal optic disc margin (nasal points 1–3; nasal peripapillary area), 1 point at the subfovea, 6 points at 900-µm intervals from the fovea to the nasal and temporal areas (nasal points 4–6; temporal points 1–3; macular area), and 2 points at 2700-µm intervals from temporal point 3 (temporal points 4 and 5; peripheral area) (Figure 1). All scans were assessed by two investigators (M.S.K. and Y.K.W.). The reproducibility of the measurements was evaluated based on the coefficient of variation (CV) and intraclass correlation coefficient (ICC). Mean values of two measurements were used for the analysis.

### 2.4. Statistical Analysis

All data analyses were performed with IBM SPSS Statistics for Windows (ver. 23.0; IBM Corp., Armonk, NY, USA). The paired *t* test was used to compare mean CT between the right and left eyes. Pearson’s correlation coefficient (ρ), ICC, and CV values were obtained to determine the interocular symmetry of CT. The absolute differences in bilateral CT measurements were determined in all measurement areas. One-way analysis of variance (ANOVA) and Bonferroni correction were used to compare interocular CT differences among the nasal peripapillary, macular, and peripheral areas. Linear regression analysis was used to analyze the relationships of interocular CT differences in the nasal peripapillary, macular, and peripheral areas with various clinical factors. All interocular difference values (e.g., in SE, IOP, AL, and CT) were obtained by subtracting the left eye values from the right eye values. In all analyses, *p* < 0.05 was taken to indicate statistical significance.

## 3. Results

### 3.1. Demographics

Among a total of 154 healthy young adults without specific findings, 121 were excluded from the study due to high myopia, history of previous refractive surgery, subfoveal CT < 300 μm, unilateral uncomplicated pachychoroid, absence of pachyvessel and inner choroidal attenuation, etc. Thus, the final study population consisted of 33 subjects with bilateral uncomplicated pachychoroid (22 men and 11 women) and an average age of 27.55 ± 2.74 years. SE, IOP, and AL were not significantly different between the right and left eyes (all *p* > 0.05) (Table 1).

### 3.2. Symmetry of Choroidal Thickness at Different Measurement Points

**Figure 2:**
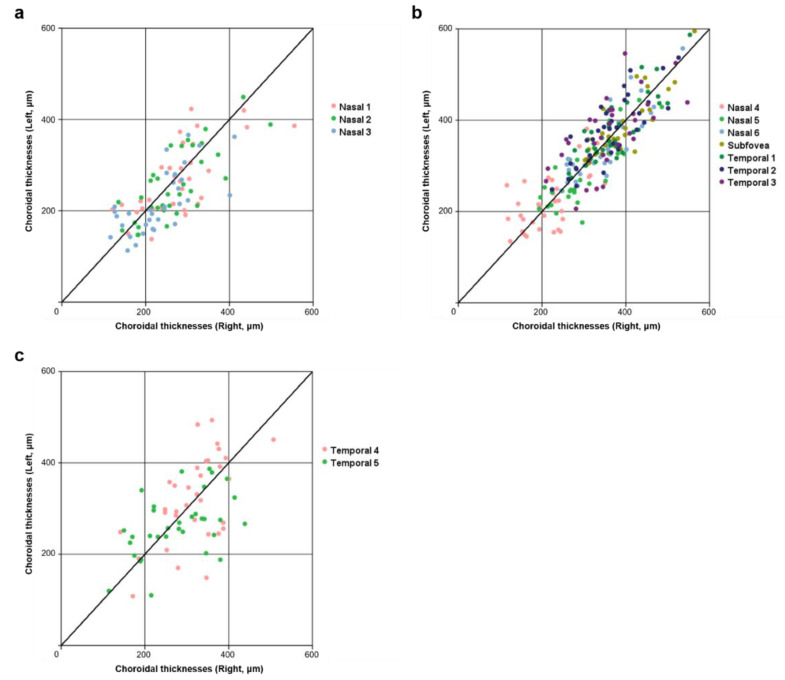
Scatter plots showing bilateral CT in uncomplicated pachychoroid subject in the nasal peripapillary (**a**), macular (**b**), and peripheral (**c**) areas. In the nasal peripapillary area (**a**), the scatter plot indicates weaker correlations than in the macular area (**b**), but stronger correlations than in the peripheral area (**c**) between right and left eye CT. CT, choroidal thickness.

The CT measurements obtained by two different investigators (M.S.K. and Y.K.W.) showed excellent reproducibility (all ICCs > 0.9 and all CVs < 10%). The mean values of subfoveal CT were 384.14 ± 67.20 μm (minimum-maximum, 302.50–564.00 μm) in the right eye and 380.36 ± 71.23 μm (minimum-maximum, 305.50–595.00 μm) in the left eye. Table 2 summarizes the mean values of the bilateral CT measurements and interocular symmetry. The average CT measurements of the right and left eyes in the nasal peripapillary area (nasal points 1–3), macular area (nasal points 4–6, subfovea, temporal points 1–3), and peripheral area (temporal points 4 and 5) showed no statistically significant differences (all *p* > 0.05). In general, larger interocular correlation coefficients (ρ) were associated with larger ICC values and smaller CV values; the reverse relation was also observed. The interocular correlation coefficients of all points in the macular area (except nasal point 4 and temporal point 3; ρ = 0.577 and 0.667, respectively) were higher than those in the nasal peripapillary area. In addition, the interocular correlation coefficients of all points in the nasal peripapillary area were higher than those in the peripheral area (Figure 2).

### 3.3. Differences in CT Measurements by Points and Area

**Figure 3:**
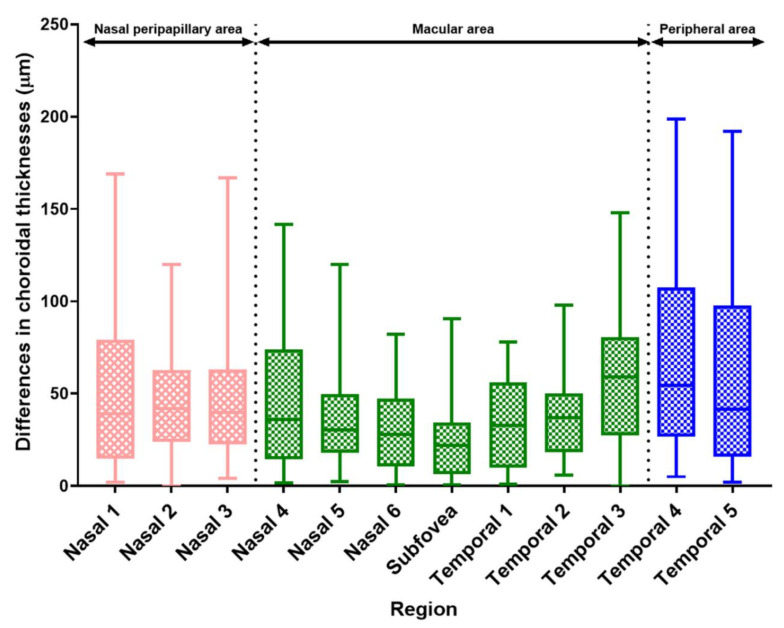
Box and whisker plots (2.5–97.5 percentile) of absolute interocular differences in CT according to region. CT differences were generally greater in the nasal peripapillary and peripheral areas than in the macular area. CT: choroidal thickness.

Table 3 shows the average absolute CT difference between the two eyes, measured at each of the 12 points. Similar to the symmetry results, the mean CT difference at all points in the macular area (except temporal point 3) was smaller than that at all points in the nasal peripapillary area. Furthermore, the mean CT difference was smaller at all points in the nasal peripapillary area than at any point in the peripheral area (Figure 3).

The mean absolute difference in CT increased gradually from the macular to the nasal peripapillary and peripheral areas (38.42 ± 28.59, 48.17 ± 34.60, and 63.36 ± 50.54 μm, respectively; *p* < 0.001, one-way ANOVA). In the Bonferroni post hoc test, the interocular absolute CT difference between the nasal peripapillary and peripheral areas was statistically significant (*p* = 0.018). The interocular absolute CT difference between macular and temporal areas was also statistically significant (*p* < 0.001). The absolute interocular CT difference between the nasal peripapillary and macular area showed a near significance (*p* = 0.058) (Table 4).

### 3.4. Clinical Factors Associated with Differences in Interocular CT According to Area

Simple linear regression analysis was performed to analyze the relationships of interocular CT differences (right eye−left eye) in three different areas (nasal peripapillary, macular, and peripheral areas) with other clinical factors (e.g., age, sex, SE, IOP, and AL) (Table 5). In terms of macular area, the interocular CT difference showed a significant negative correlation with the interocular AL difference (*β* = −6.226 ± 3.025, *p* = 0.048). In the nasal peripapillary and peripheral areas, the difference in CT was not significantly related to any clinical factor (all *p* > 0.10).

## 4. Discussion

In this study, we compared bilateral CT values among several points in the macular, nasal peripapillary, and peripheral areas using wide-field SS-OCT in subjects with uncomplicated pachychoroid. The degree of symmetry was generally high in the macular area (except nasal point 4 and temporal point 3) and low in the nasal peripapillary and peripheral areas. The interocular difference in CT increased gradually from the macular area to the nasal peripapillary and temporal areas (*p* < 0.001, one-way ANOVA), and the interocular difference in CT between the macular and nasal peripapillary area showed a near significance (*p* = 0.058) according to the post hoc Bonferroni test. This was assumed to be due to the low interocular symmetry of CT for nasal point 4 and temporal point 3 in the macular area.

According to a search of the PubMed database, no studies have analyzed interocular symmetry and differences in CT in uncomplicated pachychoroid patients. In particular, there have been no wide-field SS-OCT studies on the interocular symmetry of CT in the nasal peripapillary and peripheral areas. Most previous studies on the interocular symmetry of CT were mainly confined to foveal and parafoveal areas in healthy subjects rather than those with uncomplicated pachychoroid [2,24,25,26,27]. Therefore, this study is important in that it is the first to compare the interocular symmetry of CT among the nasal peripapillary, peripheral, and macular (foveal and parafoveal) areas using wide-field (16-mm) SS-OCT in subjects with uncomplicated pachychoroid.

Most of the studies mentioned above using Early Treatment Diabetic Retinopathy Study (ETDRS) maps or other methods reported that interocular CT in foveal and parafoveal areas showed high correlation coefficients and ICC values (ρ > 0.8, ICC > 0.9) [26,27,28]. Similarly, in the present study, CT measurements showed relatively high agreement (ρ > 0.8, ICC > 0.85) at the subfoveal, nasal 5, nasal 6, temporal 1, and temporal 2 points, which correspond to the center and inner ring (3-mm diameter area centered on the fovea) of the ETDRS map. However, CT measurements showed relatively low agreement at nasal point 4 and temporal point 3 (ρ = 0.577 and 0.667, respectively), corresponding to the outer ring of the ETDRS map. It is difficult to determine the reason for the low interocular symmetry of CT at these points. However, there have been several studies related to this issue. In the study by Chen et al. (mean (min, max) subfoveal CT = 334 (172, 568) μm in the right eye and 333 (133, 555) μm in the left eye) [26], the correlation coefficient for CT in the temporal area 3 mm from the fovea was 0.490, which was the lowest value among all areas measured in their study. In addition, other reports [29,30] posited that peripapillary CT variability could result from the presence of watershed zones, primarily near the optic disc [31]. The very low CT correlation coefficient for nasal point 4 compared to nasal point 5 in this study could be attributable to watershed zones.

To measure CT in the nasal peripapillary and peripheral areas in subjects with uncomplicated pachychoroid, wide-field imaging is required. Several studies measured peripheral choroidal CT using wide-field imaging [23,29,30,32,33]. However, to our knowledge, this is the first study to investigate interocular differences in CT in nasal peripapillary and peripheral areas in subjects with uncomplicated pachychoroid. In this study, the correlation coefficients of CT in the nasal peripapillary and peripheral areas were lower than in the macular area (except for nasal point 4 and temporal point 3), thus demonstrating that CT symmetry in the nasal peripapillary and peripheral areas is generally lower than in the macular area. However, the reason for these differences is not clear. In our previous study [23], CT measurements were compared between pachychoroid and “normochoroid eyes” using wide-field SS-OCT. Even in pachychoroid eyes, pachyvessels were sometimes absent from the nasal peripapillary and peripheral areas, leading to smaller than expected CT values. This may partly explain the greater interocular CT variation in nasal peripapillary and peripheral areas compared to the macular area.

CT is known to be affected by multiple factors. In this study, we also analyzed the relationships of interocular differences of CT with other clinical factors. In the macular area, interocular CT and AL differences had a significant negative correlation, i.e., the choroid in the macular area becomes thinner as the AL increases, and vice versa. However, in the nasal peripapillary and peripheral areas, no such correlations were seen. Thus, AL may have a greater effect on CT in the macular area than in the nasal peripapillary and peripheral areas, as suggested by previous studies [23,30]. Other factors, such as age, sex, IOP, and SE, also showed no relationships with interocular CT differences. However, only young adults were included in our study, and the age range was narrow (24–35 years; mean age = 27.55 years), which limited the generalizability of the findings. Age is known to be related to CT, but further research including detailed subgroup analyses is needed on this.

This study had some other limitations. CTs were measured manually, which can lead to measurement inaccuracy. However, the ICC and CV values showed good reproducibility, suggesting that any inaccuracy was minimal. Nevertheless, obtaining automatic CT measurements via software may be useful. We did not analyze other factors that may affect CT, such as mean arterial pressure or diurnal variation. Despite these limitations, this was the first study to compare CT among macular, nasal peripapillary, and peripheral areas in subjects with uncomplicated pachychoroid. In addition, the wide-field (16-mm) SS-OCT modality captured images without time lag, thus minimizing errors caused by time differences. Moreover, the image quality was higher, and the CT measurements were more precise in this study compared to other studies using SD-OCT or EDI-OCT. This study clearly demonstrated that interocular CT variation can occur in nasal peripapillary and peripheral areas.

In conclusion, interocular CT generally showed bilateral symmetry in our patients with uncomplicated pachychoroid, although this differed among areas. In addition, only interocular CT and AL differences were significantly correlated in the macular area; there were no significant associations for any other clinical factor. This suggests that the interocular CT difference in nasal peripapillary and peripheral areas is due to anatomical variation alone, rather than other clinical factors. Physicians should be aware of the possibility of interocular CT differences; when an uncomplicated pachychoroid patient exhibits an abnormal CT difference, it is important to perform detailed examinations to identify the factors, including other ophthalmic diseases, which may be responsible.

## Figures and Tables

**Table 1:** Baseline characteristics of participants.

Characteristic		*p*-Value
Number of patients (no. of eyes)	33 (66)	N/A
Age (mean ± SD, years)	27.55 ± 2.74	N/A
Sex (male/female)	22/11	N/A
BCVA (mean ± SD, logMAR) *		0.670
Right	−0.02 ± 0.05	
Left	−0.03 ± 0.06	
Spherical equivalent (mean ± SD, diopters) *		0.988
Right	−2.43 ± 2.00	
Left	−2.42 ± 2.01	
Intraocular pressure (mean ± SD, mmHg) *		0.233
Right	15.52 ± 3.03	
Left	16.39 ± 2.89	
Axial length (mean ± SD, mm) *		0.927
Right	24.64 ± 1.05	
Left	24.61 ± 1.10	

* Comparison between right and left eyes using the paired *t* test. BCVA, best-corrected visual acuity; logMAR, logarithm of the minimum angle of resolution; SD, standard deviation.

**Table 2:** Comparison of choroidal thickness between the right and left eyes at 12 measurement points.

	Points	Choroidal Thickness (μm) (mean ± SD)		*p*-Value *	Measure of Symmetry		
		Right Eye	Left Eye		Interocular Correlation (ρ) (All, *p* < 0.01)	ICC (All, *p* < 0.001)	CV
Nasal peripapillary area	Nasal 1	277.64 ± 87.39	268.86 ± 77.63	0.446	0.685	0.815	13.52
	Nasal 2	268.89 ± 81.61	257.08 ± 78.36	0.232	0.766	0.862	12.95
	Nasal 3	232.56 ± 73.46	217.68 ± 68.22	0.130	0.701	0.699	15.34
Macular area	Nasal 4	218.63 ± 66.06	221.58 ± 61.92	0.769	0.577	0.752	14.75
	Nasal 5	294.79 ± 71.08	284.95 ± 70.26	0.199	0.850	0.898	8.93
	Nasal 6	346.77 ± 71.04	341.77 ± 72.15	0.473	0.909	0.917	6.43
	Subfovea	384.14 ± 67.20	380.36 ± 71.23	0.537	0.842	0.926	4.85
	Temporal 1	375.33 ± 70.22	380.36 ± 71.23	0.483	0.919	0.910	6.11
	Temporal 2	364.27 ± 62.61	377.90 ± 73.84	0.010	0.873	0.891	7.63
	Temporal 3	358.05 ± 78.09	371.26 ± 78.76	0.253	0.667	0.791	11.59
Peripheral area	Temporal 4	318.21 ± 73.61	320.58 ± 96.21	0.871	0.635	0.693	15.70
	Temporal 5	278.15 ± 86.25	263.14 ± 68.13	0.287	0.530	0.644	15.98

* Comparison of choroidal thickness between the right and left eyes by paired *t* test. CV, coefficient of variation; ICC, interclass correlation coefficient; ρ. Pearson’s correlation coefficient; SD, standard deviation.

**Table 3:** Differences in absolute choroidal thickness between the right and left eyes at 12 measurement points.

Region		Difference in Choroidal Thickness (μm)	
		Mean ± SD	95% CI
Nasal peripapillary area	Nasal 1	51.29 ± 40.45	38.73–66.32
	Nasal 2	47.15 ± 30.87	37.42–59.07
	Nasal 3	46.06 ± 32.58	36.41–57.71
Macular area	Nasal 4	44.94 ± 34.36	33.14–56.45
	Nasal 5	35.62 ± 25.47	26.99–44.29
	Nasal 6	31.67 ± 23.62	23.49–40.12
	Subfovea	27.11 ± 23.28	19.77–35.01
	Temporal 1	33.15 ± 23.44	25.94–41.00
	Temporal 2	39.94 ± 25.70	31.03–48.69
	Temporal 3	56.52 ± 33.68	45.62–68.62
Peripheral area	Temporal 4	66.12 ± 48.07	49.81–84.60
	Temporal 5	60.59 ± 52.78	42.52–79.18

CI, confidence interval; SD, standard deviation.

**Table 4:** Interocular difference in CT among the nasal peripapillary, macular, and peripheral areas.

		Region			Statistical Significance			
		Nasal Peripapillary Area	Macular Area	Peripheral Area	*p* *	*p* ^†^	*p* ^‡^	*p* **
Difference in CT (μm)	Mean ± SD	48.17 ± 34.60	38.42 ± 28.59	63.36 ± 50.54	<0.001	0.058	0.018	<0.001
	95% CI	41.27–55.07	34.53–40.98	51.11–78.36				

CI, confidence interval; CT, choroidal thickness; SD, standard deviation. * Among the nasal peripapillary area, macular, peripheral areas (one-way analysis of variance). ^†^ Between the nasal peripapillary and macular areas (Bonferroni post hoc test). ^‡^ Between the nasal peripapillary and temporal areas (Bonferroni post hoc test). ** Between the macular and temporal areas (Bonferroni post hoc test).

**Table 5:** Simple regression analysis of the relationships of clinical factors with the mean interocular difference in choroidal thickness by area.

	Simple Regression Analysis (β ± SD)					
Area	Nasal Peripapillary Area	*p*	Macular Area	*p*	Peripheral Area	*p*
Age	−5.058 ± 3.169	0.121	−0.185 ± 2.121	0.931	−5.235 ± 3.940	0.194
Sex (male = 0, female = 1)	9.538 ± 18.645	0.618	−1.792 ± 12.237	0.885	−18.966 ± 23.123	0.418
Interocular difference						
Intraocular pressure	−0.975 ± 1.908	0.613	−1.447 ± 1.205	0.239	−0.259 ± 2.354	0.913
Spherical equivalent	1.832 ± 2.816	0.520	3.069 ± 1.739	0.087	4.823 ± 3.374	0.163
Axial length	7.275 ± 4.839	0.143	−6.226 ± 3.025	0.048	−3.933 ± 6.118	0.525

Interocular differences were obtained by subtracting right eye values from left eye values. SD, standard deviation.

## Data Availability

The data that support the findings of this study are available from the corresponding author upon reasonable request.
